# Invasive Plant Species Distribution Is Structured by Soil and Habitat Type in the City Landscape

**DOI:** 10.3390/plants10040773

**Published:** 2021-04-15

**Authors:** Ilona Szumańska, Sandra Lubińska-Mielińska, Dariusz Kamiński, Lucjan Rutkowski, Andrzej Nienartowicz, Agnieszka Piernik

**Affiliations:** Department of Geobotany and Landscape Planning, Faculty of Biology and Veterinary Sciences, Nicolaus Copernicus University in Toruń, 87-100 Toruń, Poland; ilona.szumanska@gmail.com (I.S.); sanlub@doktorant.umk.pl (S.L.-M.); daro@umk.pl (D.K.); lrutkow@umk.pl (L.R.); anienart@umk.pl (A.N.)

**Keywords:** alien species, expansion, IAS, invasion, urban invasions, Central Europe, Toruń

## Abstract

Invasive alien species (IAS) is a global problem that largely relates to human activities and human settlements. To prevent the further spread of IAS, we first need to know their pattern of distribution, to determine which constitutes the greatest threat, and understand which habitats and migration pathways they prefer. Our research aimed to identify the main vectors and distribution pattern of IAS of plants in the city environment. We checked the relations between species distribution and such environmental factors as urban soil type and habitat type. We applied data on IAS occurrence (collected in the period 1973–2015) in 515 permanent plots with dimensions of 0.5 × 0.5 km and analyzed by direct ordination methods. In total, we recorded 66 IAS. We found a 27% variance in the IAS distribution pattern, which can be explained by statistically significant soil and habitat types. The most important for species distribution were: river and alluvial soils, forests and related rusty soils, and places of intensive human activities, including areas of urbisols and industriosols. Our results provide details that can inform local efforts for the management and control of invasive species, and they provide evidence of the different associations between natural patterns and human land use.

## 1. Introduction

Invasive species have been an important research topic for scientists from around the world for several decades [1,2]. Therefore, there is a certain subjectivity among scientists when using this term [3]. For the purposes of this article, we adopt the definition according to Tokarska-Guzik et al. [1], in line with that proposed by the International Union for Conservation of Nature (IUCN) [4] and by the Global Invasive Species Program [5]. Invasive alien species (IAS) means a non-native species that has established itself in ecosystems or habitats that are either natural, semi-natural, or man-made, e.g., some agroecosystems [6]. Importantly, these species are a trigger of changes that threaten native biodiversity, economy, and/or human health [1,4].

The invasion of alien species of plants into a natural ecosystem or habitat is a complex problem. Invasive species can affect the soil and its microorganisms, and thus native species of plants as well, often leading to their displacement [7]. This may affect not only the biodiversity of the flora but also the fauna of the affected area [8,9]. IAS can pose a threat to both natural ecosystems and crops, human health, and some branches of industry, such as tourism [10]. Fighting the spread of IAS often requires considerable financial resources [11].

Research on invasive species has been going on for centuries and is becoming more intense as both awareness and the incidence of the problem rise [12]. Many years of research have shown that one of the important vectors for spreading invasive plant species is humans—their activities and the infrastructure that they create [13,14]. People can act on purpose or unintentionally; e.g., seeds can be transferred on the clothes and shoes of unwitting people, just as on animal hair or bird feathers [15,16].

Humans are not the only vector for spreading invasive plants, but they are often one of the contributing factors—man-made surfaces as concrete and asphalt amplify the natural spreading ability of some species [17]. This is caused by the phenomenon of secondary wind dispersal on these impervious surfaces within road corridors, especially present in cities.

Cities, like other human settlements, are specific places for introducing alien species [18]. IAS in cities have very good conditions to spread. First of all, large amounts of alien species are introduced into cities for ornamental purposes, e.g., in city parks, along communication routes, and into home gardens [19]. Alien species do very well in cities due to the presence of urban heat islands and numerous disturbances that facilitate the spread and development of plants [18]. The presence of the mentioned communication routes, ruderal habitats, as well as harbors, water reservoirs, and rivers is important [20]. IAS appearing in cities can do a lot of damage as they threaten both infrastructure (roots damage walls or pavements) and human health (causing sensitization and allergies) [21]. However, primarily, they displace native species [22,23]. Invasive species in cities pose a threat not only to the cities themselves but also to the landscape outside the city, where they can spread through the transport corridors or watercourses flowing through cities, wind, and zoochory [24,25,26].

According to Gulezian and Nyberg [22], there is usually no relationship between the type of habitat in a city and the invasion of specific species. However, the type of land use influences the number of invasive species [27]. There is also an obvious negative correlation between species abundance and the presence of an impervious surface [22]. Moreover, results by Štajerova et al. [18] demonstrate that a great amount of IAS variation in the city can be explained by spatial predictors and the species cover of invasive species can decrease with an increasing proportion of urban greenery but increase with road margins, ruderal sites, and railway sites. This implies that habitat structure in the city landscape can be crucial for IAS distribution. Understanding IAS behavior in the urban environment is of crucial importance to future landscape management and activities against invasions and threats to the natural ecosystem.

Therefore, the aim of our research was to identify the main vectors and patterns of IAS distribution in the city landscape. We performed our research in a medium-size city on the Vistula river bank in central Poland. We hypothesized that (a) there is a relation between IAS pattern and habitat or soil type, and (b) habitat or soil type can drive IAS richness within the city. Accordingly, the research was conducted in terms of land cover and habitat preferences of recorded IAS in the context of soil and habitat types within the city limits.

## 2. Results

### 2.1. Invasive Species Flora

We recorded 66 plant species recognized as IAS in Poland [1], of which we selected 31 with full data available to analyze their distribution (Table S1). The most frequent were *Erigeron canadensis* L. and *Acer negundo* L., present in 98–97% of plots; next, *Robinia pseudoacacia* L. and *Galinsoga parviflora* Cav., noted in over 80% of plots; then, *Solidago gigantea* Aiton, *Prunus serotina* Ehrh., *Cannabis sativa* L., *Lycium barbarum* L., and *Parthenocissus inserta* (A. Kern.) Fritsch in over 50% of plots. In 20–40% of plots we recorded *Quercus rubra* L., *Helianthus tuberosus* L., *Juglans regia* L., *Rumex confertus* Willd., *Echinocystis lobata* (Michx.) Torr. & A. Gray, *Bidens frondosa* L., *Xanthium albinum* (Widd.) Scholz & Sukopp and *Elodea canadensis* Michx. The least frequent were *Eragrostis multicaulis* Steud., *Impatiens parviflora* DC., *Elodea nuttallii* (Planch.) H.St.John, *Impatiens glandulifera* Royle, *Fraxinus pennsylvanica* Marshall, *Reynoutria japonica* Houtt., *Asclepias syriaca* L., *Bunias orientalis* L., *Erechtites hieracifolia* (L.) Raf., which were recorded in between 1% and 15% of plots, and *Ailanthus altissima* (Mill.) Swingle, *Clematis vitalba* L., *Celtis occidentalis* L., *Ambrosia artemisiifolia* L., *Reynoutria sachalinensis* (F. Schmidt) Nakai, which were noted in less than 1% of plots. Most of the invaders came from North America (ca. 55%) followed by Asia and south and east Europe (ca. 32% combined). In this group, we have seven trees: *A. negundo*, *R. pseudoacacia*, *Q. rubra*, *F. pensylvanica*, and *C. occidentalis* from North America, *J. regia*, and *A. altissima* from Asia, and one north-American shrub, *P. serotina*.

### 2.2. Invasive Species Distribution Patterns

#### 2.2.1. Species—Soil-Type Relations

**Figure 1:**
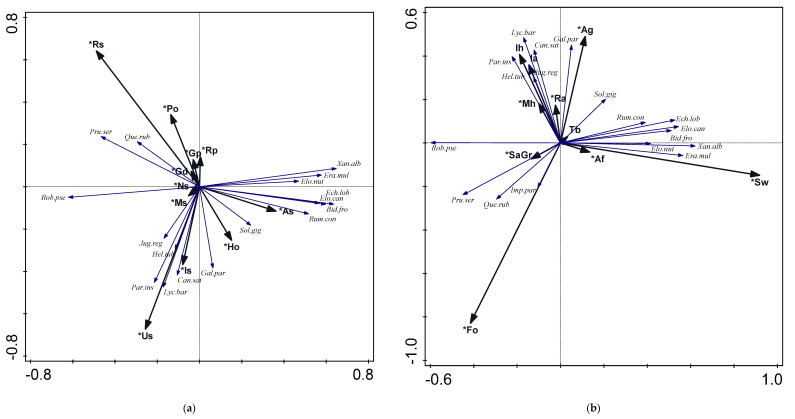
Results of redundancy analysis (RDA) with forward selection and Monte Carlo permutation test: (**a**) relation between invasive plant species distribution and urban soil types, species over 3% fit are presented in ordination space of axis I and II; (**b**) relation between invasive plant species distribution and habitat types, species over 3% fit are presented in ordination space of axis I and II. * significant variables (*p* < 0.05). Abbreviations of urban soil types and habitat types as in Table 1. Abbreviations of plant names consist of the first three letters of the genus name and the first three letters of the species name, e.g., Que.rub—*Quercus rubra* L., Pru.ser—*Prunus serotina* Ehrh., full list in Table S1.

Searching for invasive flora distribution patterns, first, we checked the relationship between the presence of species and the urban soil type. Results of direct ordination (RDA), forward selection, and Monte Carlo significance test demonstrated that areas of rusty soils (Rs) and urbisols (Us) accounted for the largest amount of variance in invasive plant species distribution pattern, i.e., together ca. 12% (Table 1). Among recorded species, the presence of *Q. rubra*, *P. serotina*, and, partly, *R. pseudoaccacia* was related to Rs, whereas *C. sativa*, *L. barbarum*, *H. tuberosus*, *P. inserta,* and *J. regia* were related to Us (Figure 1a). Podzols (Po), industrisols (Is), alluvial soils (As), and hortisols (Ho) were also statistically significant in the species–soil-type relation model (Table 1). *R. confertus*, *E. lobata*, *B. frondosa*, *X. albiuum*, *E. multicaulis*, *Elodea canadensis*, and *E. nuttallii* were present in plots with the domination of As. The occurrences of *G. parviflora* and partly *S.gigantea* were related to the presence of Ho. The remainder of the soil types were also significant in the model but explained less than 1% of the species variance.

#### 2.2.2. Species—Habitat-Type Relations

In the pattern of invasive plant species distribution determined by habitat types, the most important were river (Sw) and forest areas (Fo), which explained respectively 11 and ca. 7% of the species distribution variance (Table 1). *R. confertus*, *E. lobata*, *B. frondosa*, *X. albinum*, *E. multicaulis*, *Elodea canadensis*, and *E. nuttallii*, described previously as being related to alluvial soils (As), were at the same time related to the river (Sw), which was, of course, to be expected (Figure 1b). The presence of *I. parviflora* and *Q. rubra* was connected with forest areas (Fo), whereas *P. serotina* was additionally associated with open sandy areas (SaGr). A relatively large group of species (i.e., *G. parviflora*, *C. sativa*, *L. barbarum*, *H. tuberosus*, *P. inserta,* and *J. regia*) was significantly correlated with allotment gardens, shrubs and grasslands (Ag), multifamily housing areas (Mh), and ruderal areas (Ra) (Table 1, Figure 1b).

**Figure 2:**
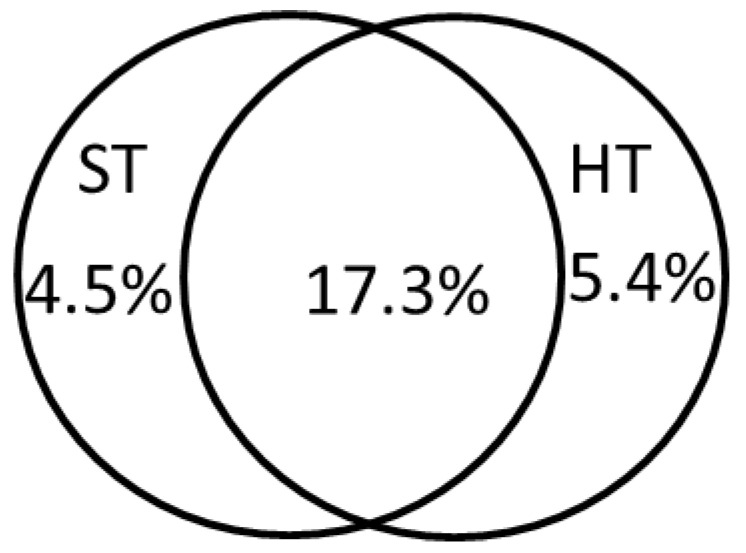
Partitioning of the variance in invasive species data into the contributions of two subsets of environmental variables, which denote soil types (ST) and habitat types (HT) and shared portion by these two groups.

The division of variance in species distribution into two groups of factors (i.e., soil type and habitat type) revealed that all these factors explained ca. 27.1% of the total variation, but soil-type factors explained ca. 4.5% and habitat type 5.4%, while they shared relatively large responsibility for the variation in species data, amounting to ca. 17.3% (Figure 2).

#### 2.2.3. Invasive Species Richness

**Figure 3:**
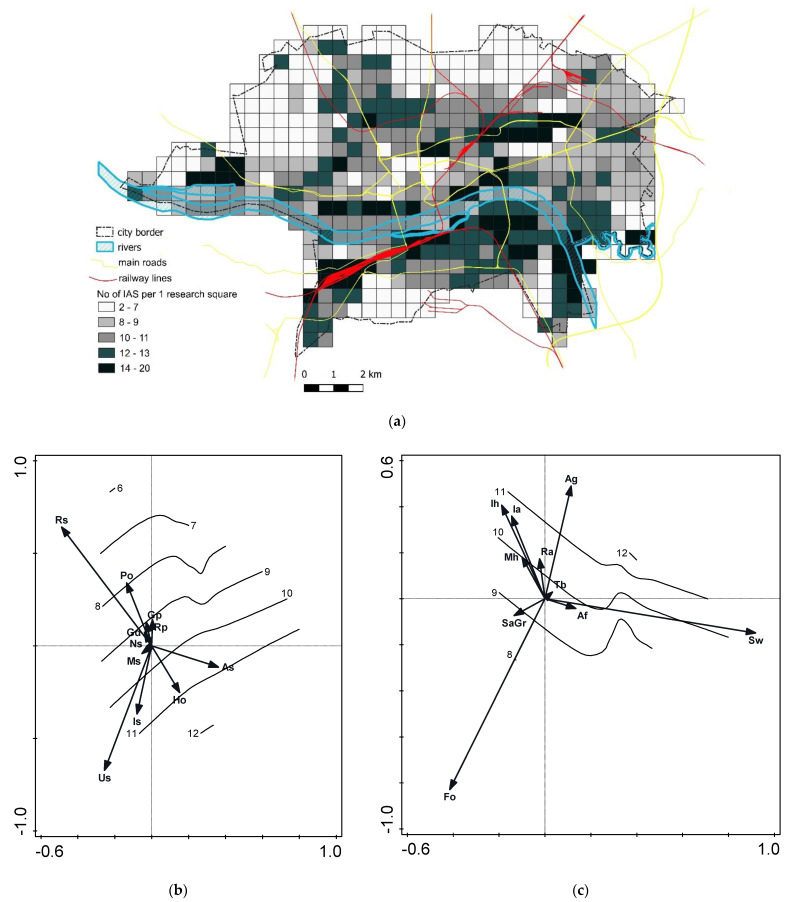
Species richness models: (**a**) spatial IAS number in permanent plots, only species noted within city borders are included; (**b**) loess model of species number (isolines) in the gradient of soil types based on redundancy analysis (RDA); (**c**) loess model of species number (isolines) in the gradient of soil types based on redundancy analysis (RDA). Abbreviations of urban soil types and habitat types as in Table 1.

The results of invasive species distribution within the monitored plot demonstrated their richness in selected regions of the city (Figure 3a). Analysis of the distribution of these areas showed that they are related to the river cutting through the city and to the main roads and railway lines. We noted a maximum of 20 species out of 31 in some plots. Northern border areas of the town (covered by pine forest monoculture) were relatively poor in invasive species, reaching from two up to seven different plant invaders. The largest richness of invasive species covered about 14 km^2^ (10% of the total area), whereas the largest area of ca. 37 km^2^ was inhabited by two IAS (27.4% of the total area). The most common were *Erigeron canadensis* and *A. negundo*, as already mentioned. Species number increases on urbisols (Us), industriosols (Is), hortisols (Ho), and alluvial soils (As) (Figure 3b), in areas of allotment gardens, shrubs, and grasslands (Ag), and in the river valley (Sw) (Figure 3c).

## 3. Discussion

We recorded 66 alien invasive plant species in the city of Toruń which is 4.5% of the total number of Toruń’s flora, estimated at 1466 taxa. Compared to currently available data from other European cities, this is quite a small number. A comparable number was observed in, for example, the similarly sized city of Hradec Králové (the Czech Republic)—42 invasive neophytes [18]. However, in the much larger city of Bratislava (the capital of Slovakia), in ruderal areas alone, 26 invasive taxa were recorded [28]. Data collected by La Sorte et al. [29] (Appendix S1) has shown that in other European capital cities even more invasive species have been found, e.g., Vienna (Austria)—141 (10.0% of city flora), Prague (Czech Republic)—138 (13.54%), Berlin (Germany)—121 (12.78%), Dublin (Ireland)—107 (19.7%), Rome (Italy)—213 (16.92%), Warsaw (Poland)—95 (17.96%). All these capitals, except Dublin, are cities at least twice the size of Toruń. Although this relationship has not been investigated, it seems that the city area size does not have a decisive influence on the number of invasive plant species present there. For example, 55 more invasive species were found in Wrocław (Poland), as compared to Warsaw, which is a little over twice the size [29].

Research by La Sorte et al. [29] showed also that the flora of European cities has a greater percentage of archaeophytes and invasive species compared to cities outside Europe—mainly in North America. Additionally, the studies of Lososová et al. [30] show that the urban flora of Central Europe consists of ca. 54% native species, and similar numbers of archaeophytes and neophytes, i.e., 24% and 22%, respectively. Therefore the number of new and old arrivals there is almost comparable.

According to results presented here, the most common in the city of Toruń was *Erigeron canadensis* and *A. negundo*, which are present in almost all plots. Already published research shows that the invasiveness of specific species can vary dependent on latitude, and invasive plants do best in conditions similar to those prevailing where they come from. For example, *Erigeron canadensis* is clearly influenced by temperature [31]. This species is common throughout Poland. Research in the Warta River valley showed that *Erigeron canadensis* has increased its range since the 1980s, spreading through the river valley, as well as within ruderal areas [32]. In general, *Erigeron canadensis* is one of the most successful invaders outside North America [33,34]. The second most common species, *A. negundo*, is also common not only in Toruń but in almost the whole of Poland and other European countries [35,36]. This species was introduced for ornamental purposes. Within cities, it occupies mainly ruderal sites. It is also common in riparian ecosystems, where it poses a great threat. It spreads similarly to *Erigeron canadensis*—along river valleys [32,37,38]. Both species were not related to any soil type or habitat type, as they were present in almost all plots.

Other common recorded species were *R. pseudoacacia* and *G. parviflora*—noted in over 80% of plots. *R. pseudoacacia* is a species that has been studied for years for its invasiveness, both in Poland and Europe as well as globally [37,39,40,41,42,43]. People intentionally introduced this plant to new areas for various reasons, e.g., as an ornamental plant, to prevent soil erosion, or as a source of nectar. Currently, this persistent species is a great threat to the plant richness and stability of the species composition of forests, in dry and semi-arid grasslands and alluvial areas. It also appears in cities and post-industrial areas. *G. parviflora* is common in almost the whole of Poland and in many regions across the world [44]. The species spreads mainly in anthropogenic habitats and is considered a common weed in gardens and fields. Due to the loss in crops it can cause, it is analyzed in economic terms, but also as a threat to local biodiversity [37,45]. This problem related to the spreading of *G. parviflora* has affected many countries, and not only in Europe. The appearance of *G. parviflora* is often associated with changes in the agriculture of a given area [46,47]. Research is being carried out to find crop species capable of suppressing the invasion of this and other weeds in agroecosystems, e.g., sweet potato [48].

Our results have confirmed our hypothesis, that there is a relationship between species distribution pattern and environmental factors because ca. 27% of the variance in IAS distribution models has been explained by statistically significant soil and habitat types. It is worth emphasizing that over half of this variance was related to both of these groups of factors simultaneously. The most important for species distribution were river (Sw) and alluvial soils (As), forests (Fo) and related rusty soils (Rs), and places of intensive human activities including areas of urbisols (Us), industriosols (Is), and hortisols (Ho) such as allotment gardens, shrubs, and grasslands (Ag), multifamily housing areas (Mh) and ruderal areas (Ra).

The group of species related to alluvial soils (As) in the river valley (Sw) has already been reported as spreading along rivers. *R. confertus* was noted in Poland at the end of the 19th century in the Bug valley [49] and was described as being able to spread along river valleys [50,51]. *E. lobata* was introduced in Poland for ornamental purposes and was often cultivated in allotments, from where it spread to natural habitats mainly within willow and poplar riparian forests on river and lake banks [32,37]. This species has also spread widely along rivers and streams in Bashkortostan Republic (Russia), where its presence causes even osier-beds to dry out [52]. The spread of *B. frondosa* along rivers is confirmed by other studies: in Poland along the Odra river from Germany, then along the Bug and Vistula river valleys [37]; in Russia, e.g., on the banks of the Rybinsk Reservoir located on the Upper Volga river [53]; in disturbed riverside habitats in the upper stream of the Iset’ river [54]; and in the Bashkortostan Republic [52]. Similarly, the spread of *X. albinum* is mainly related to human-disturbed habitats: along two large rivers (the Vistula and the Bug) in Poland [37]; along the Elbe river in Germany [55]; and in the flood plains of rivers, in moist riverbed habitats and coastal sands in Russia (Bashkortostan Republic) [52]. Moreover, Poland’s largest river and valley, the Vistula, is also described as the main corridor for the spread of *E. multicaulis* in Poland [37,56]. Interestingly, the species is known as a weed in rice fields, where it poses an economic problem: the plants can survive even when their roots are completely flooded [57,58,59].

We found also that some species occur mainly in forests (Fo) on rusty soils (Rs). *Q. rubra* and *P. serotina* were intentionally introduced in forests of European countries and beyond, e.g., in Italy in temperate deciduous woodland patches of the Po plain, in the Lombardy region, or Ukraine, France, Germany, and the British Isles [60,61]. The soils occupied by these species are therefore mainly conditioned by places of their introduction. This explains the presence of *P. serotina* on sandy soil because the species was introduced into pine monocultures [37] that grow over such habitats. These species pose a threat to biodiversity, protected areas, landscape, and economy [37,62,63]. *I. parviflora*, which we found to also be related to city forests, spread in a slightly different way in Europe. One hypothesis states that it spread as an escapee from botanical gardens to ruderal habitats from where it entered forests (mainly deciduous). Alternatively, it could have made its way to Europe by accident due to sea travelers [37]. The success of *I. parviflora* is related to, among other things, its great ability to survive in various types of forests [64]. *I. parviflora*, *Q. rubra* and *P. serotina* are among the most invasive and most common IAS in Polish national parks [65].

The group of species related to allotment gardens, shrubs and grasslands (Ag), multifamily housing areas (Mh), and ruderal areas (Ra), i.e., *G. parviflora*, *C. sativa*, *L. barbarum*, *H. tuberosus*, *P. inserta,* and *J. regia*, demonstrates the places where IAS have the potential to spread inside and outside the city landscape. People who behave irresponsibly in their home gardens and allotments may even unintentionally increase the range and accelerate the invasion of invasive species. This is the case when, for example, they remove their bio-waste and leave it in nearby forests [66]. In this way, species can get into favorable habitats and quickly spread outside cities, e.g., in forests surrounding cities. Even arboretums and botanical gardens can pose a threat that alien plant species will spread from their areas [67], the more so in the case of city parks.

Such common species as *R. pseudoacacia*, and partly *S. gigantea* have almost no preferences in their distribution pattern, because of their large tolerance to environmental conditions. However, within city borders *S. gigantea* was related to gardening areas of hortisols, as it is still cultivated for ornamental purposes (e.g., [37]).

Finally, we also confirmed our second hypothesis—that richness of different IAS may be related to soil and habitat type. We can interpret that the most important for species number was the presence of vacant environmental niches (disturbed areas along river banks [Sw, As], housing areas [Mh], and ruderal places [Ra]), sources of diaspores (allotments and home gardens [Ag]), and transport routes (river valley [Sw] and roads), along which we found higher numbers of invasive species. There are favorable conditions for growth in these places, but also the possibility of transport across city borders, e.g., along roads or rivers e.g., [68,69]. Such dependence of IAS number on landscape structure has been reported by, among others, Manier et al. [70] and Štajerova et al. [18]. Moreover, Štajerova et al. [18] noticed that the number of invasive species decreased with an increasing proportion of urban greenery, which was also presented by our results.

According to Wagner et al. [71], many of the species that occur in Toruń are among the most common alien plant species in European woodlands, i.e., *I. parviflora*, *P. serotina*, *R. pseudoacacia*, *Q. rubra*, *I. glandulifera*, *S. gigantea*, *B. frondosa*, *A. negundo*, and *Erigeron canadensis*. This implies that if invasive species are not monitored sufficiently, their presence may have different consequences for both species diversity in cities and the surrounding landscape.

All plant species analyzed in this paper belong to the invasive group. Each of them has the appropriate mechanisms to succeed in new territories. Due to research in other areas, many lists of invasive species have been created for individual smaller and larger regions. Popular on a global scale is ‘100 of the world’s worst invasive alien species: a selection from the global invasive species database’ [72]. None of the IAS species recorded in the investigated city is on this list. However, this does not mean that the species found here include none that pose a threat to the biological diversity of cities and their adjacent areas. That is why our results provide details that can inform local efforts for management and control of invasive species, and they provide evidence of the different associations between natural patterns and human land use.

## 4. Materials and Methods

### 4.1. The Research Area

The research was carried out within administrative borders of the city of Toruń (Central Poland, 52°58′–53°04′ N and 18°32′–18°43′ E). The city is located on the left and right bank of the Vistula river. The city covers 115 km^2^. The distance between the extreme points to the east and west is ca. 19 km, and 12 km north and south. The city has 191,227 inhabitants. The climate of Toruń is characterized by low mean values of precipitation, amounting to 522.5 mm. The average annual temperature in the years 1951–2000 ranged from 6.0 °C (1956) to 9.9 °C (2000). The warmest month is July (mean temperature of 18.1 °C), and the coldest, January (−2.2 °C). The vegetation period lasts for 218 days [73,74].

### 4.2. Data Collection

**Figure 4:**
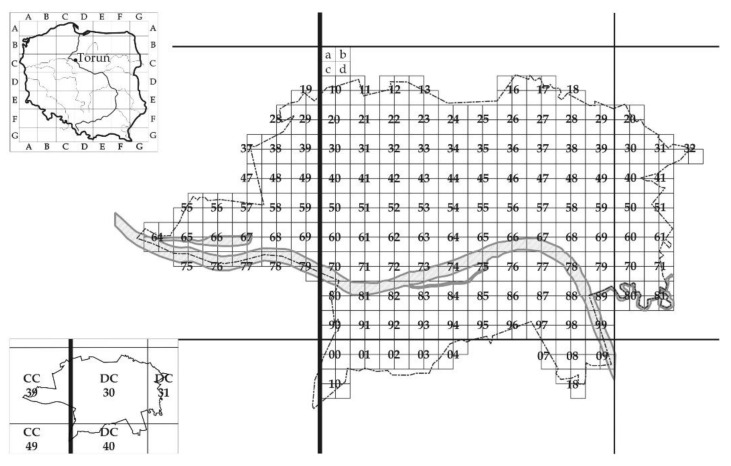
Location and area of data collection in the city of Toruń in a grid of ATPOL squares (0.5 × 0.5 km).

Data about IAS were collected from 1973 through 2015 within general inventory research of flora in the city of Toruń. Available data on species distribution (presence/absence) were placed in a grid of squared plots based on the ATPOL (Atlas of Poland) system [75], which is a common floristic research method in Poland [76], currently based on World Geodetic System WGS 84. Based on this system we divided the research area into 515 squares of 0.5 × 0.5 km (Figure 4). In each square, we recorded plant species recognized as alien invasive for Poland according to Tokarska-Guzik et al. [1]. The species names were unified according to World Flora Online [77]. For statistical analysis, we included only species with complete current distribution in ATPOL plots.

Environmental data regarding habitat type were based on a generalization of satellite data [78] and synthesis of soils in each of the plots (Figures S1 and S2). The vector map of soils [79] was generalized into a raster of 0.5 × 0.5 km grid of species data plots. In the same way, a vector map of habitat type was generalized into a raster. For the generalization, we used the ArcGIS software, version 9.3, and the methods described by Adamska and Juśkiewicz [78]. In that way, we obtained eleven types of dominant soil types in 515 plots (Figure S1): Rs—rusty soils, Us—urbisols, Is—industriosols, Po—podzols, As—alluvial soils, Rp—replantosols, Ho—hortisols, Ms—mucky soils, Ns—necrosols, Gp—gleyic podzols, Gd—garbage dump (nomenclature followed Hulisz et al. [80]) and ten local habitat types (Figures S2 and S3): Fo—forests, Ag—allotment gardens, shrubs and grasslands, Ra—ruderal areas, SaGr—sands and gravels, Af—arable fields, Ow—open water, Mh—multifamily housing areas, Ia—industrial areas, Ih—individual housing areas, and Tb—tenement buildings.

### 4.3. Data Analyses

Based on invasive species data in each square, the map of the concentration of invasive species in the city was generated in ArcGIS software, version 9.3. Distribution patterns of invasive species in the gradient of soil types and land-cover classes were analyzed using the direct ordination method and CANOCO 5.0 software [81]. We applied two runs of redundancy analysis (RDA) together with a forward selection procedure and Monte Carlo permutation test to assess the relative importance of each environmental variable and its statistical significance [81,82]. In the first run, species data were used as dependent variables and soil data as an independent. In the second RDA run, the dependent variables remained the same, but habitat types were applied as the independent variables. In both runs, the loess model was applied with *span* = 1 and robust fitting algorithm to obtain the model of IAS number in the gradient of independent variables. To assess the relative importance of soil types compared to habitat types we applied a species variance partitioning procedure into these two groups [83].

## 5. Conclusions

Our findings proved that variance in the IAS distribution pattern can be explained by statistically significant soil and habitat types. The most important for species distribution were: river and alluvial soils, forests and related rusty soils, and places of intensive human activities, including areas of urbisols and industriosols. More detailed not only qualitative but also quantitative research on IAS distribution and environmental factors could help in the future in better understanding urban invasions in a diverse city environment. However, our results now provide details that can inform local efforts for management and control of invasive species, and they provide evidence of the different associations between natural patterns and human land use.

## Figures and Tables

**Table 1:** Results of forward selection and Monte Carlo permutation test demonstrating relative importance and statistical significance of soil types and habitat types in IAS distribution pattern.

Soil-Type Effects				Habitat-Type Effects			
Variable	V %	F	*p*	Variable	V %	F	*p*
Rs	6.6	36.4	0.002	Sw	11.0	63.5	0.002
Us	5.4	31.2	0.002	Fo	6.8	42.3	0.002
Is	2.8	16.7	0.002	Af	1.7	11.1	0.002
Po	2.4	14.9	0.002	Ag	1.2	7.9	0.002
As	1.3	7.9	0.002	SaGr	0.9	5.8	0.002
Rp	1.6	10.3	0.002	Mh	0.6	4.1	0.002
Ho	1.9	12.5	0.002	Ra	0.4	2.3	0.02
Ms	0.8	5.4	0.002	Tb	0.3	1.9	0.084
Ns	0.5	3.6	0.002	Ia	0.2	1.2	0.254
Gp	0.5	3.2	0.008	Ih	0.2	1.2	0.334
Gd	0.5	3.3	0.004				

Soil types: Rs—rusty soils, Us—urbisols, Is—industriosols, Po—podzols, As—alluvial soils, Rp—replantosols, Ho—hortisols, Ms—mucky soils, Ns—necrosols, Gp—gleyic podzols, Gd—garbage dump. Habitat types: Fo—forests, Ag—allotment gardens, shrubs and grasslands, Ra—ruderal areas, SaGr—sands and gravels, Af—arable fields, Sw—surface water, Mh—multifamily housing areas, Ia—industrial areas, Ih—individual housing areas, and Tb—tenement buildings. Other abbreviations: V%—percentage of IAS variance explained in the model, F—Fisher parameter, *p*—significance level.

## Data Availability

The datasets used and/or analyzed during the current study are available from the corresponding author on reasonable request.

## Supplementary Materials

The following are available online at https://www.mdpi.com/article/10.3390/plants10040773/s1, Figure S1: Choropleth map of natural and anthropogenic soil types in the city of Toruń, Figure S2: Choropleth map of habitat types in the city of Toruń, Figure S3: The selected habitat types within the borders of the city of Toruń, Table S1: Invasive plant species recorded in the city of Toruń, Poland.

## References

[1] Tokarska-Guzik B., Dajdok Z., Zając M., Zając A., Urbisz A., Danielewicz W., Hołdyński C. (2014). Rośliny Obcego Pochodzenia w Polsce ze Szczególnym Uwzględnieniem Gatunków Inwazyjnych.

[2] Ramírez-Albores J.E., Badano E.I., Flores J., Flores-Flores J.L., Yáñez-Espinosa L. (2019). Scientific literature on invasive alien species in a megadiverse country: Advances and challenges in Mexico. NeoBiota.

[3] Colautti R.I., MacIsaac H.J. (2004). A neutral terminology to define ‘invasive’ species. Divers. Distrib..

[4] IUCN (World Conservation Union) IUCN Guidelines for the Prevention of Biodiversity Loss Caused by Alien Invasive Species. Proceedings of the SSC Invasive Species Specialist Group, Approved by the 51st Meeting of the IUCN Council.

[5] Independent Evaluation Group (2009). The Global Invasive Species Program. Global Program Review.

[6] Perrino E.V., Calabrese G. (2018). Endangered segetal species in southern Italy: Distribution, conservation status, trends, actions and ethnobotanical notes. Genet. Resour. Crop Evol..

[7] Lazzaro L., Bolpagni R., Buffa G., Gentili R., Lonati M., Stinca A., Acosta A.T.R., Adorni M., Aleffi M., Allegrezza M. (2020). Impact of invasive alien plants on native plant communities and Natura 2000 Habitats: State of the art, gap analysis and perspectives in Italy. J. Environ. Manag..

[8] Jordan N.R., Larson D.L., Huerd S.C. (2007). Soil modification by invasive plants: Effects on native and invasive species of mixed-grass prairies. Biol. Invasions.

[9] Litt A.R., Cord E.E., Fulbright T.E., Schuster G.L. (2014). Effects of Invasive Plants on Arthropods. Conserv. Biol..

[10] EPPO (European and Mediterranean Plant Protection Organization) (2009). Data sheet on Invasive Alien Plants: *Heracleum mantegazzianum*, *Heracleum sosnowskyi* and *Heracleum persicum*. Bull. OEPP.

[11] Hoffmann B.D., Broadhurst L.M. (2016). The economic cost of managing invasive species in Australia. NeoBiota.

[12] Simberloff D. (2013). Invasive Species: What Everyone Needs to Know.

[13] Kowarik I., von der Lippe M., Nentwig W. (2007). Pathways in Plant Invasions. Biological Invasions.

[14] Bhowmik P.C., Inderjit (2005). Characteristics, significance, and human dimension of global invasive weeds. Invasive Plants: Ecological and Agricultural Aspects.

[15] Auffret A.G., Cousins S.A.O. (2013). Humans as Long-Distance Dispersers of Rural Plant Communities. PLoS ONE.

[16] Coughlan N.E., Kelly T.C., Jansen M.A.K. (2014). Mallard duck (*Anas platyrhynchos*)—Mediated dispersal of Lemnaceae: A contributing factor in the spread of invasive *Lemna minuta*?. Plant Biol..

[17] Kowarik I., von der Lippe M. (2011). Secondary wind dispersal enhances long-distance dispersal of an invasive species in urban road corridors. NeoBiota.

[18] Štajerová K., Šmilauer P., Brůna J., Pyšek P. (2017). Distribution of invasive plants in urban environment is strongly spatially structured. Landsc. Ecol..

[19] Shackleton C.M., Shackleton R.T. (2016). Knowledge, perceptions and willingness to control designated invasive tree species in urban household gardens in South Africa. Biol. Invasions.

[20] Aronson M.F.J., Patel M.V., O’Neill K.M., Ehrenfeld J.G. (2017). Urban riparian systems function as corridors for both native and invasive plant species. Biol. Invasions.

[21] Pejchar L., Mooney H.A. (2009). Invasive species, ecosystem services and human well-being. Trends Ecol. Evol..

[22] Gulezian P.Z., Nyberg D.W. (2010). Distribution of invasive plants in a spatially structured urban landscape. Landsc. Urban Plan..

[23] Francis R.A., Chadwick M.A. (2015). Urban invasions: Non-native and invasive species in cities. Geography.

[24] von der Lippe M., Kowarik I. (2008). Do cities export biodiversity? Traffic as dispersal vector across urban-rural gradients. Divers. Distrib..

[25] Cutway H.B., Ehrenfeld J.G. (2009). Exotic plant invasions in forested wetlands: Effects of adjacent urban land use type. Urban Ecosyst..

[26] Padayachee A.L., Irlich U.M., Faulkner K.T., Gaertner M., Procheş Ş., Wilson J.R.U., Rouget M. (2017). How do invasive species travel to and through urban environments?. Biol. Invasions.

[27] Godefroid S., Ricotta C. (2018). Alien plant species do have a clear preference for different land uses within urban environments. Urban Ecosyst..

[28] Rendeková A., Mičieta K., Hrabovský M., Eliašová M., Miškovic J. (2019). Effects of invasive plant species on species diversity: Implications on ruderal vegetation in Bratislava City, Slovakia, Central Europe. Acta Soc. Bot. Pol..

[29] La Sorte F.A., Aronson M.F.J., Williams N.S.G., Celesti-Grapow L., Cilliers S., Clarkson B.D., Dolan R.W., Hipp A., Klotz S., Kühn I. (2014). Beta diversity of urban floras among European and non-European cities. Glob. Ecol. Biogeogr..

[30] Lososová Z., Chytrý M., Tichý L., Danihelka J., Fajmon K., Hájek O., Kintrová K., Láníková D., Otýpková Z., Řehořek V. (2012). Biotic homogenization of Central European urban floras depends on residence time of alien species and habitat types. Biol. Conserv..

[31] Wang C., Zhou J., Liu J., Xiao H., Wang L. (2017). Functional traits and reproductive allocation strategy of *Conyza canadensis* as they vary by invasion degree along a latitude gradient. Pol. J. Environ. Stud..

[32] Dyderski M.K., Jagodziński A.M. (2016). Patterns of plant invasions at small spatial scale correspond with that at the whole country scale. Urban Ecosyst..

[33] Yan H., Feng L., Zhao Y., Feng L., Zhu C., Qu Y., Wang H. (2020). Predicting the potential distribution of an invasive species, *Erigeron canadensis* L., in China with a maximum entropy model. Glob. Ecol. Conserv..

[34] Shah M.A., Callaway R.M., Shah T., Houseman G.R., Pal R.W., Xiao S., Luo W., Rosche C., Reshi Z.A., Khasa D.P. (2014). *Conyza canadensis* suppresses plant diversity in its nonnative ranges but not at home: A transcontinental comparison. New Phytol..

[35] Straigytė L., Cekstere G., Laivins M., Marozas V. (2015). The spread, intensity and invasiveness of the *Acer negundo* in Riga and Kaunas. Dendrobiology.

[36] Erfmeier A., Böhnke M., Bruelheide H. (2010). Secondary invasion of *Acer negundo*: The role of phenotypic responses versus local adaptation. Biol. Invasions.

[37] Tokarska-Guzik B. (2005). The Establishment and Spread of Alien Plant Species (Kenophytes) in the Flora of Poland.

[38] Dyderski M.K., Gdula A.K., Jagodziński A.M. (2015). “The rich get richer” concept in riparian woody species—A case study of the Warta River Valley (Poznań, Poland). Urban For. Urban Green..

[39] Sabo A.E. (2000). *Robinia pseudoacacia* invasions and control in North America and Europe. Restor. Reclam. Rev..

[40] Benesperi R., Giuliani C., Zanetti S., Gennai M., Lippi M.M., Guidi T., Nascimbene J., Foggi B. (2012). Forest plant diversity is threatened by *Robinia pseudoacacia* (black-locust) invasion. Biodivers. Conserv..

[41] Vítková M., Müllerová J., Sádlo J., Pergl J., Pyšek P. (2017). Black locust (*Robinia pseudoacacia*) beloved and despised: A story of an invasive tree in Central Europe. For. Ecol. Manag..

[42] Lazzaro L., Mazza G., d’Errico G., Fabiani A., Giuliani C., Inghilesi A.F., Lagomarsino A., Landi S., Lastrucci L., Pastorelli R. (2018). How ecosystems change following invasion by *Robinia pseudoacacia*: Insights from soil chemical properties and soil microbial, nematode, microarthropod and plant communities. Sci. Total Environ..

[43] Dyderski M.K., Jagodziński A.M. (2019). Seedling survival of *Prunus serotina* Ehrh., *Quercus rubra* L. and *Robinia pseudoacacia* L. in temperate forests of Western Poland. For. Ecol. Manag..

[44] Damalas C.A. (2008). Distribution, biology, and agricultural importance of *Galinsoga parviflora* (Asteraceae). Weed Biol. Manag..

[45] Węgrzynek B., Tokarska-Guzik B., Trueman I.C., Cohn E., Rabitsch W., Essl F., Klingenstein F. (2008). *Galinsoga* species in Poland: History of spread and habitat preferences of two successful alien weeds. Biological Invasions—From Ecology to Conservation.

[46] Stadler J., Mungai G., Brandl R. (1998). Weed invasion in East Africa: Insights from herbarium records. Afr. J. Ecol..

[47] Török K., Botta-Dukát Z., Dancza I., Németh I., Kiss J., Mihály B., Magyar D. (2003). Invasion Gateways and Corridors in the Carpathian Basin: Biological Invasions in Hungary. Biol. Invasions.

[48] Shen S., Xu G., Li D., Jin G., Liu S., Clements D.R., Yang Y., Rao J., Chen A., Zhang F. (2019). Potential Use of Sweet Potato (*Ipomoea batatas* (L.) Lam.) to Suppress Three Invasive Plant Species in Agroecosystems (*Ageratum conyzoides* L., *Bidens pilosa* L., and *Galinsoga parviflora* Cav.). Agronomy.

[49] Eichler B., Łapczyński K. (1892). Korespondencja do Wszechświata. Wszechświat.

[50] Stosik T. (2008). *Rumex confertus* Willd-biologia a ekspansja gatunku. Ann. Univ. Mariae Curie-Skłodowska E Agric..

[51] Raycheva T. (2011). *Rumex confertus* (Polygonaceae) in the Bulgarian flora. Bot. Serb..

[52] Abramova L.M., Golovanov Y.M. (2018). Invasions of Alien Plant Species in the South Urals: Current State of the Problem. KnE Life Sci..

[53] Maltseva S.Y., Bobrov A.A. (2017). Alien species of vascular plants in the Rybinsk Reservoir (Upper Volga, Russia). Russ. J. Biol. Invasions.

[54] Ronzhina D.A. (2017). Distribution, competitive ability, and seed production of *Bidens frondosa* L. in the Middle Urals. Russ. J. Biol. Invasions.

[55] Sukopp H., Starfinger U., Edwards K., Kowarik I., Williamson M. (1998). On the study of anthropogenic plant migrations in central Europe. Plant Invasions: Ecological Mechanisms and Human Responses.

[56] Sudnik-Wójcikowska B., Guzik J. (1996). The spread and habitats of *Eragrostis pilosa* (Poaceae) in the Vistula valley. Fragm. Florist. Geobot. Pol..

[57] Glatkova G., Surek H., Andov D., Andreevska D., Pacanoski Z. (2019). *Eragrostis pilosa* (L.) P. Beauv. a New Invasive and Economically Important Weed in the Rice Fields in the Kocani Region. Int. J. Innov. Approaches Agric. Res..

[58] Carloto B.W., Buriol G.A., Dornelles S.H.B., Trivisiol V.S., Peripolli M., Escobar O.S. (2019). Morphological and phenological responses of *Eragrostis plana* Nees and *Eragrostis pilosa* (L.) P. Beauv. plants subjected to different soil moisture conditions. Planta daninha.

[59] Carloto B.W., Escobar O. (2020). dos S.; Trivisiol, V.S.; Peripolli, M.; Pivetta, M.; Posser, T.; Barreto, E.P.M.; Dornelles, S.H.B. Response of *Eragrostis plana* and *Eragrostis pilosa* (L.) P. Beauv. submitted on flooded soil. Acta Sci. Biol. Sci..

[60] Gentili R., Ferrè C., Cardarelli E., Montagnani C., Bogliani G., Citterio S., Comolli R. (2019). Comparing Negative Impacts of *Prunus serotina*, *Quercus rubra* and *Robinia pseudoacacia* on Native Forest Ecosystems. Forests.

[61] Dyderski M.K., Chmura D., Dylewski Ł., Horodecki P., Jagodziński A.M., Pietras M., Robakowski P., Woziwoda B. (2020). Biological Flora of the British Isles: *Quercus rubra*. J. Ecol..

[62] Stanek M., Piechnik Ł., Stefanowicz A.M. (2020). Invasive red oak (*Quercus rubra* L.) modifies soil physicochemical properties and forest understory vegetation. For. Ecol. Manag..

[63] Dyderski M.K., Jagodziński A.M. (2021). How do invasive trees impact shrub layer diversity and productivity in temperate forests?. Ann. For. Sci..

[64] Florianová A., Münzbergová Z. (2018). Drivers of natural spread of invasive *Impatiens parviflora* differ between life-cycle stages. Biol. Invasions.

[65] Bomanowska A., Adamowski W., Kirpluk I., Otręba A., Rewicz A. (2019). Invasive alien plants in Polish national parks—threats to species diversity. PeerJ.

[66] Šipek M., Šajna N. (2020). Public opinions and perceptions of peri-urban plant invasion: The role of garden waste disposal in forest fragments. Manag. Biol. Invasions.

[67] Ferus P., Hoťka P., Košútová D., Konôpková J. (2020). Invasions of alien woody plant taxa across a cluster of villages neighbouring the Mlyňany Arboretum (SW Slovakia). Folia Oecologica.

[68] Hansen M.J., Clevenger A.P. (2005). The influence of disturbance and habitat on the presence of non-native plant species along transport corridors. Biol. Conserv..

[69] Christen D.C., Matlack G.R. (2008). The habitat and conduit functions of roads in the spread of three invasive plant species. Biol. Invasions.

[70] Manier D.J., Aldridge C.L., O’Donnell M., Schell S.J. (2014). Human Infrastructure and Invasive Plant Occurrence Across Rangelands of Southwestern Wyoming, USA. Rangel. Ecol. Manag..

[71] Wagner V., Chytrý M., Jiménez-Alfaro B., Pergl J., Hennekens S., Biurrun I., Knollová I., Berg C., Vassilev K., Rodwell J.S. (2017). Alien plant invasions in European woodlands. Divers. Distrib..

[72] Lowe S., Browne M., Boudjelas S., De Poorter M. (2000). 100 of the World’s Worst Invasive Alien Species: A Selection from the Global Invasive Species Database.

[73] Wójcik G., Marciniak K., Andrzejewski L., Weckwerth P., Burak S. (2006). Klimat. Toruń i Jego Okolice. Monografia Przyrodnicza.

[74] Przybylak R., Uscka-Kowalkowska J., Araźny A., Kejna M., Kunz M., Maszewski R. (2017). Spatial distribution of air temperature in Toruń (Central Poland) and its causes. Theor. Appl. Clim..

[75] Zając A. (1978). Atlas of distribution of vascular plants in Poland (ATPOL). Taxon.

[76] Komsta Ł. (2016). Rewizja matematyczna siatki geobotanicznej ATPOL—Propozycja algorytmów konwersji współrzędnych. [ATPOL geobotanical grid revisited—A proposal of coordinate conversion algorithms. Ann. Univ. Mariae Curie-Skłodowska E Agric..

[77] World Flora Online. http://www.worldfloraonline.org/.

[78] Adamska E., Juśkiewicz W. (2018). Visualisation of the influence of habitat on lichen occurrence, Toruń, Poland. J. Maps.

[79] Bednarek R., Jankowski M., Andrzejewski L., Weckwerth P., Burak S. (2006). Gleby. Toruń i Jego Okolice—Monografia Przyrodnicza.

[80] Hulisz P., Charzyński P., Greinert A. (2016). Urban soil resources of medium-sized cities in Poland: A comparative case study of Toruń and Zielona Góra. J. Soils Sediments.

[81] ter Braak C.J.F., Šmilauer P. (2012). Canoco Reference Manual and User’s Guide: Software for Ordination (Version 5.0).

[82] Jongman R.H.G., ter Braak C.J.F., van Tongeren O.F.R. (1995). Data Analysis in Community and Landscape Ecology.

[83] Šmilauer P., Lepš J. (2014). Multivariate Analysis of Ecological Data Using CANOCO 5.

