# Supplementary material for: Invasive Plant Species Distribution Is Structured by Soil and Habitat Type in the City Landscape
Source: Plants (Basel). 2021 Apr 15;10(4):773. doi: 10.3390/plants10040773 (PMC8071169; doi:10.3390/plants10040773)
Supplement: Supplementary file 1 [file plants-10-00773-s001.zip › plants-1177880-supplementary.pdf]

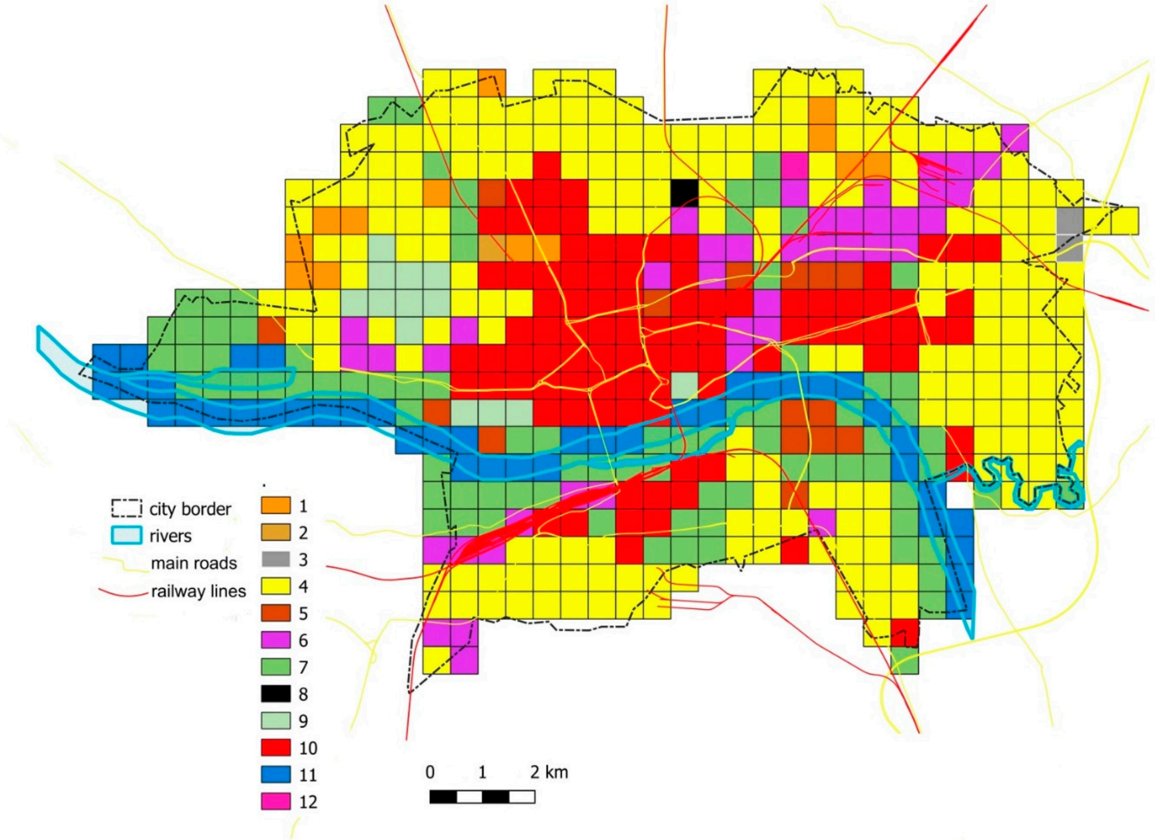

Figure S1. Choropleth map of natural and anthropogenic soil types in the city of Toruń (according to: [79]): 1—podzols, 2—gleyic podzols, 3—mucky soils, 4—rusty soils, 5—hortisols, 6—industriosols, 7—alluvial soils, 8—necrosols, 9—replantosols, 10—urbisols, 11—surface water, 12—garbage dump.

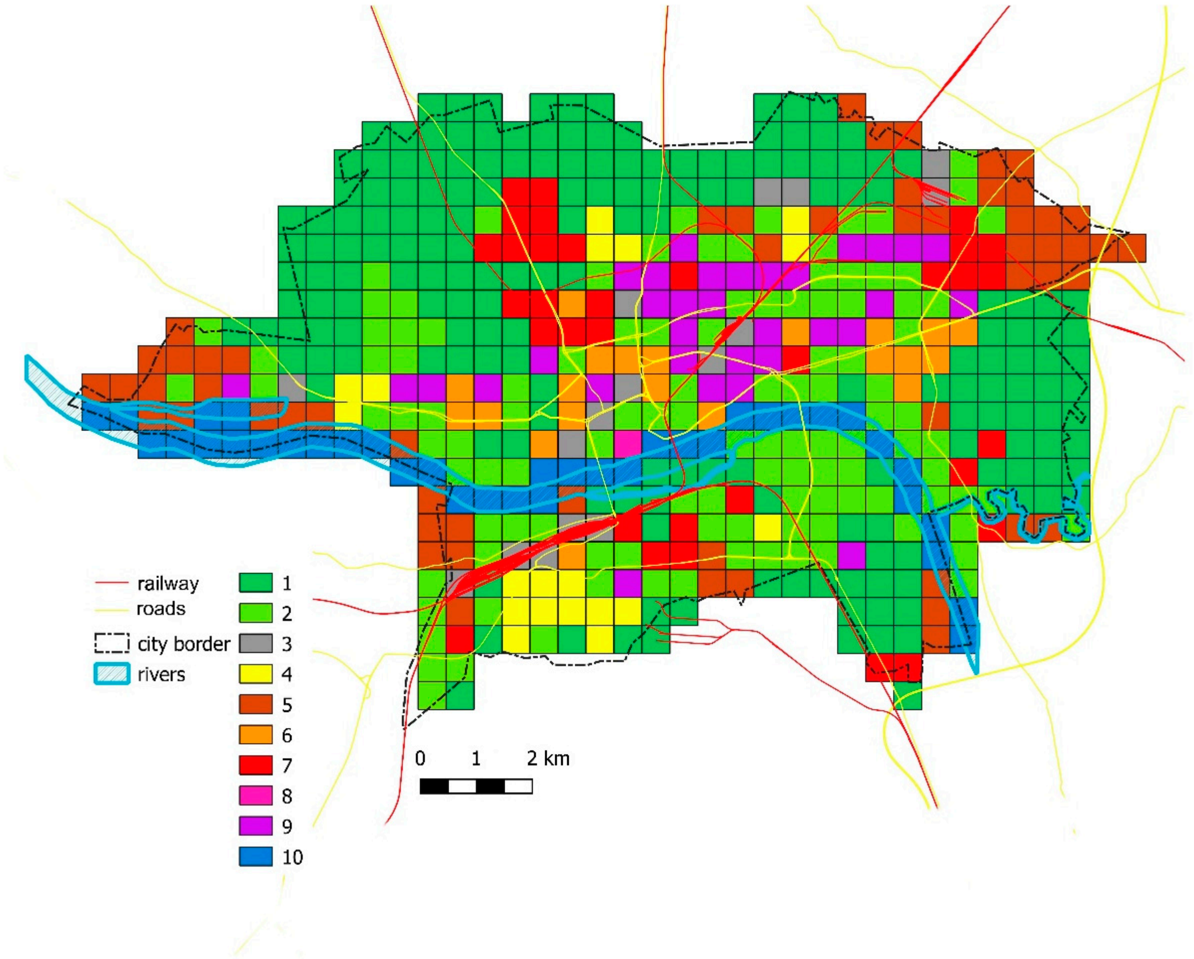

Figure S2. Choropleth map of habitat types in the city of Toruń: 1—forests, 2—shrubs, grasslands, allotment gardens, 3—ruderal areas, 4—sandy and gravel areas, 5—arable fields, 6—multifamily housing, 7—individual housing, 8—tenements, 9—industrial areas, 10 – surface water (according to: Mieczysław Kunz, Department of Geomatics and Cartography, Faculty of Earth Sciences and Spatial Management NCU in Toruń) [78].

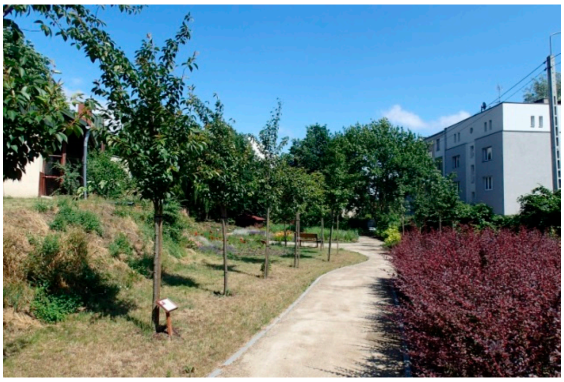

Multi-family housing area (Mh)

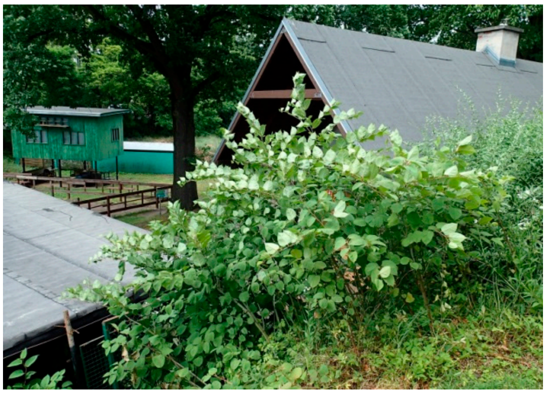

Individual housing area (Ih)

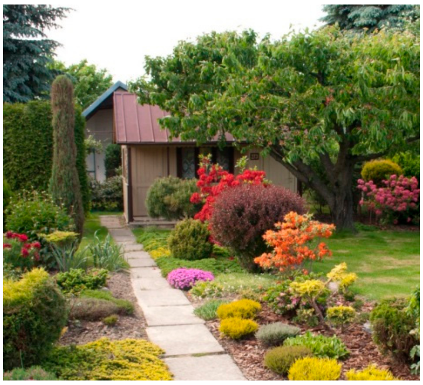

Allotment garden (Ag)

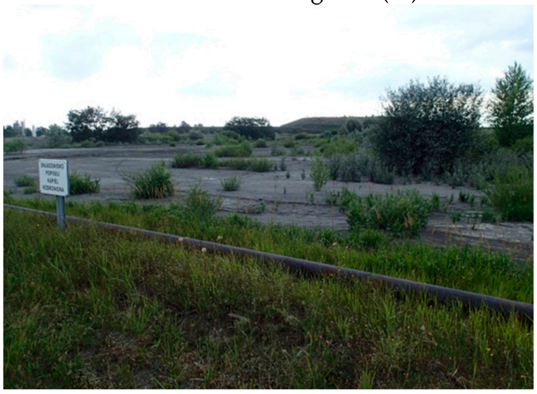

Industrial areas –landfill with mixture of ash and slag in Power Plants (Ia)

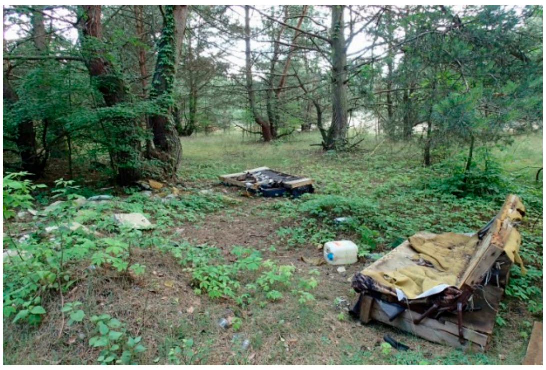

Ruderal area (Ra)

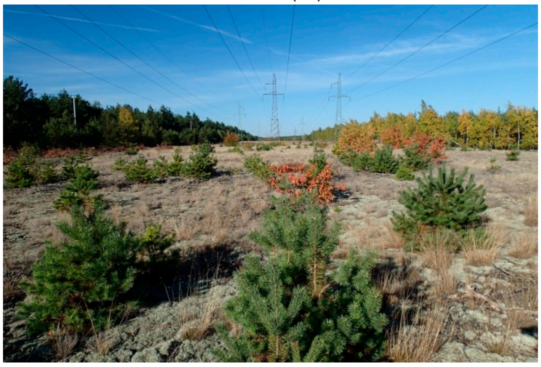

Sandy area (SaGr)

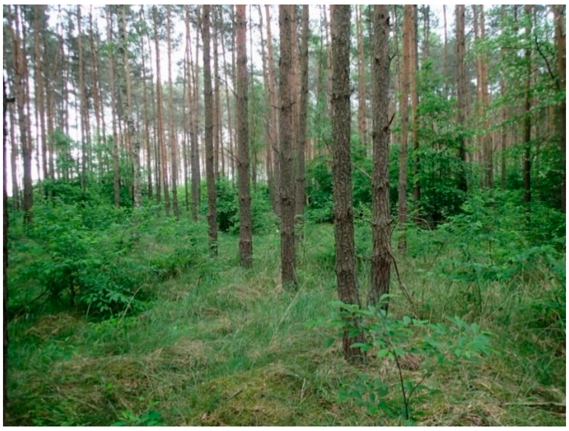

Forest (Fo)

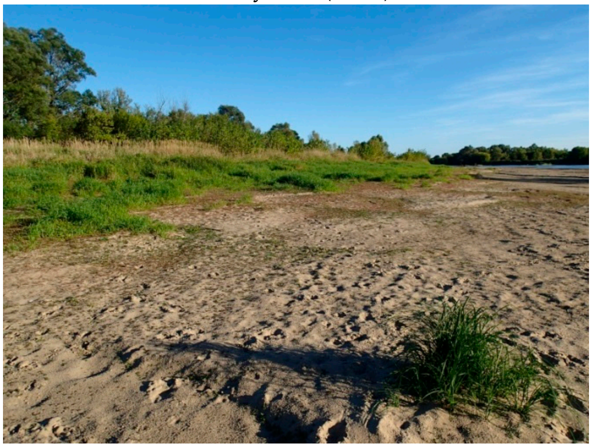

River bank area (Sw, As)

Figure S3. Selected habitat types within the borders of the city of Toruń.

Table S1. Invasive plant species recorded in the city of Toruń, Poland. Species identified as invasive according to: Tokarska-Guzik et al. [1]; species name given according to: World Flora Online [77].

Species Name	Abbreviation of Species Name	Origin	First Time Recorded	Frequency [%]
Species with full ATPOL location (included in statistical analyzes)				
Acer negundo L.	Ace.neg	North America	1808	97.3
Ailanthus altissima (Mill.) Swingle	Ail.alt	Asia E	1808	0.8
Ambrosia artemisiifolia L.	Amb.art	North America	1613	0.4
Asclepias syriaca L.	Asc.syr	North America	18th century	1.6
Bidens frondosa L.	Bid.fro	North America	1777	27.1
Bunias orientalis L.	Bun.ori	Europe SE, Asia W	1858	1.2
Cannabis sativa L. (*Cannabis sativa var. spontanea* Vavilov)	Can.sat	Asia	20th century	59.1
Celtis occidentalis L.	Cel.occ	North America	1805	0.6
Clematis vitalba L.	Cle.vit	Europe WS, Asia, Africa	1613	0.8
Erigeron canadensis L.	Eri.can	North America	1730	98.4
Echinocystis lobata (Michx.) Torr. & A. Gray	Ech.lob	North America	1937	27.3
Elodea canadensis Michx.	Elo.can	North America	1867	18.2
Elodea nuttallii (Planch.) H.St.John	Elo.nut	North America	1990	5.4
Eragrostis multicaulis Steud.	Era.mul	Europe SE, Asia W	20th century	14.1
Erechtites hieracifolia (L.) Raf.	Ere.hie	North America	1902	1.2
Fraxinus pennsylvanica Marshall	Fra.pen	North America	1805	2.5
Galinsoga parviflora Cav.	Gal.par	Central & South America	1807	82.6
Helianthus tuberosus L.	Hel.tub	North America	1730	38.4
Impatiens glandulifera Royle	Imp.gla	Central Asia	1890	2.9
Impatiens parviflora DC.	Imp.par	Central & Eastern Asia	1850	7.9
Juglans regia L.	Jug.reg	Asia SE, E	18th century	36.0
Lycium barbarum L.	Lyc.bar	Europe SE, Asia E	1847	50.0
Prunus serotina Ehrh.	Pru.ser	Central & North America	1813	72.7
Parthenocissus inserta (A.Kern.) Fritsch	Par.ins	North America	1806	49.2
Quercus rubra L.	Que.rub	North America	1806	39.7
Reynoutria japonica Houtt.	Rey.jap	Asia E	1882	2.3
Reynoutria sachalinensis (F. Schmidt) Nakai	Rey.sac	Asia E	1903	0.4
Robinia pseudoacacia L.	Rob.pse	North America	18th century	85.1
Rumex confertus Willd.	Rum.con	Europe SE, Asia W	1873	34.5
Solidago gigantea Aiton	Sol.gig	North America	1853	79.5
Xanthium albinum (Widd.) Scholz & Sukopp	Xan.alb	North America	1853	18.4

Species without full ATPOL location (excluded from statistical analyzes)		
**Species name**	**Origin**	**First time recorded**
Alopecurus myosuroides Huds.	Mediterranean, Asia SW	no data
Amaranthus retroflexus L.	Central & North America	1801
Amelanchier spicata (Lam.) K.Koch	North America	1820
Avena fatua L. s.l.	Asia SW	no data
Bromus carinatus Hook. & Arn.	North America	1911
Buddleja davidii Franch.	Asia	1911
Cornus sericea L.	North America	1811
Cotoneaster lucidus Schltdl.	Asia E	1889
Digitalis purpurea L.	Europe W	1809
Diplotaxis muralis (L.) DC.	Europe SW	1850
Echinochloa crus-galli (L.) P.Beauv.	Asia	no data
Epilobium ciliatum Raf.	North America	1917
Erigeron annuus (L.) Pers.	North America	1830
Galinsoga quadriradiata Ruiz & Pav.	America Central & South	1876

Heliopsis helianthoides subsp. scabra (Dunal) T.R.Fisher	North America	19th century
Heracleum mantegazzianum Sommier et Levier	Kaukaz	1973
Heracleum sosnowskyi Manden.	Kaukaz	1980
Hordeum murinum L.	Europe, Asia	no data
Juncus tenuis Willd.	North America	1862
Lolium multiflorum Lam.	Europe SE, Asia SW, Africa N	1837
Lupinus polyphyllus Lindl.	North America	1877
Lysimachia punctata L.	Europe SE	1870
Berberis aquifolium Pursh	North America	1839
Oxalis corniculata L.	Europe S, Asia SW, Africa, Australia	1863
Oxalis stricta L.	North America	1809
Rhus typhina L.	North America	1806
Rosa rugosa Thunb.	Asia E	19th century
Rudbeckia laciniata L.	North America	1787
Setaria pumila (Poir.) Roem et Schult.	Asia SE	no data
Setaria viridis (L.) P.Beauv.	Asia SE, Mediterranean	no data
Solidago canadensis L.	North America	1872
Symphyotrichum novi-belgii (L.) G.L.Nesom	North America	18th century
Symphyotrichum × salignum (Willd.) G.L.Nesom	North America	19th century
Veronica persica Poir.	Asia SW	1862
Vicia grandiflora Scop.	Europe S, Asia SW	1907

First time recorded—first time recorded in Poland; Frequency in permanent plots.
